# Development of microRNA-based therapy for pancreatic cancer

**DOI:** 10.1097/jp9.0000000000000029

**Published:** 2019-12

**Authors:** Andrew Fesler, Jingfang Ju

**Affiliations:** Department of Pathology, Stony Brook University, Stony Brook, NY

**Keywords:** Chemotherapy, Gemcitabine, miRNA, Pancreatic ductal adenocarcinoma, Resistance

## Abstract

Despite extensive research efforts on diagnosis and treatment, pancreatic ductal adenocarcinoma (PDAC) remains a devastating disease and the third leading cause of cancer-related death in the United States. Resistance to current therapeutic approaches is a major reason for the poor survival of pancreatic patients. In order to overcome this major challenge and improve patient outcomes, we are in desperate need of novel therapeutic approaches. PDAC chemoresistance mechanisms are complex and multifaceted. Novel therapeutics must be equipped to deal with this challenge. microRNAs (miRNAs) have emerged as strong candidates to fill this role due to their multitargeted function. miRNAs have been shown to have important roles in pancreatic cancer resistance. In this review, we summarize the recent advancement in miRNA research related to PDAC therapeutic resistance mechanisms and the potential of miRNAs as therapeutic agents for future clinical management of PDAC.

## Background

In the past several decades we have made some great progress in treating several cancers; however, there is still much more that needs to be done to have a dramatic impact on patient survival. Pancreatic ductal adenocarcinoma (PDAC) in particular remains a considerable challenge. Several factors including typically late presentation, early metastasis, and resistance to chemotherapy, all contribute to PDAC being the third deadliest cancer in the United States.^[1,2]^ While currently patients are typically treated with surgical resection, adjuvant chemotherapy, and radiotherapy, many patients experience postoperative recurrence.^[3,4]^ For the past 20 years, gemcitabine has been the standard first-line chemotherapeutic used to treat pancreatic cancer.^[5]^ Over this time, the efficacy of different therapeutic regiments based around gemcitabine, have been tested including combination of gemcitabine with several other agents such as 5-fluorouracil (5-FU), oxaliplatin, cisplatin, and capecitabine.^[6–9]^ Despite these efforts, the impact on patient survival has remained limited, due in part to the various resistance mechanism involved in PDAC cells. Some PDAC patients will have intrinsic resistance and never respond to therapy, while others will have acquired resistance that will develop during the course of treatment leading to recurrence.^[10]^ Since resistance is such a major issue for treating PDAC patients, extensive research efforts have been aimed at understanding the mechanisms involved in PDAC chemoresistance. These efforts have demonstrated that PDAC chemoresistance is multifaceted. PDAC cells have altered expression of several important genes such as KRAS, p53, and BCL-2 among others. These cells also have changes in important signaling pathways such as Notch and Hedgehog.^[10–13]^ Factors in the tumor microenvironment, secreted by tumor-associated fibroblasts, also contribute to resistance.^[14–17]^ The presence of PDAC stem cells also contributes to resistance, as these cells have several characteristics that make them highly resistant to chemotherapy. These cells are slowly dividing, making them more resistant than rapidly dividing cells. These cells also have a very plastic nature, and altered metabolism that allows them to cope with the challenge of therapy.^[18]^ The need for novel therapeutics is clear based on the poor survival for PDAC patients, only 3% for patients with metastatic disease.^[1]^ The fact that various mechanism contribute to resistance makes microRNA (miRNA) an ideal candidate to combat this challenge as their multitargeted function may be more challenging for cancer cells to develop resistance.

miRNAs are short (18–22 nucleotides) noncoding RNAs that regulate gene expression by base pairing primarily to the 3′-untranslated region of target messenger RNA (mRNA) to inhibit translation or induce mRNA degradation. Each miRNA is able to target several different target genes, allowing them to inhibit the expression of a network of targets. Depending on the target genes they inhibit, miRNAs may promote cancer progression and resistance (onco-miRNAs) or inhibit progression and resistance (tumor suppressor miRNAs). In the past decade, it has become clear that miRNAs have important roles in all cancers including PDAC. We have also come to appreciate the potential for manipulating miRNAs for therapeutic intervention.^[19]^

## miRNAs functions in PDAC resistance mechanisms

It is now understood that miRNAs have important functions in PDAC and dysregulation of their expression may play a role in cancer development as well as resistance. miRNAs regulate tumorigenesis, cell cycle control, apoptosis, proliferation, chemoresistance, invasion, and metastasis.^[19]^ There are plenty of examples of the important functions miRNAs have in PDAC resistance. Some miRNAs promote chemoresistance in PDAC. miR-21 is one of the best characterized onco-miRs in many cancers. In PDAC high expression of miR-21 has been shown to inhibit the effectiveness of gemcitabine. High expression is also associated with shorter patient survival. These effects of miR-21 are a result of its targeting phosphatase and tensin homolog, and suppression of miR-21 results in cell cycle arrest, apoptosis, and enhanced sensitivity to chemotherapy.^[20–22]^ In addition to miR-21, several other miRNAs have been shown to promote resistance in PDAC. miR-320a, promotes resistance to 5-FU in PDAC by targeting programmed cell death domain 4.^[23]^ miR-221–3p also increases PDAC resistance to 5-FU by targeting Retinoblastoma 1 (RB1). In addition to promoting chemoresistance, by targeting RB1 miR-221–3p promotes cell proliferation, migration invasion, and epithelial-to-mesenchymal transition (EMT).^[24]^ miR-106a is another miRNA that promotes chemoresistance. In the case of miR-106a, it is expressed in exosomes from cancer-associated fibroblasts.^[25]^ miR-155 also has chemoresistance-associated functions in PDAC involving exosomes. Gemcitabine treatment promotes miR-155 expression which induces increased production of exosomes and inhibition of apoptosis to promote resistance. miR-155 is also packaged in exosomes promoting resistance in nearby cells.^[26]^ In PDAC stem cells, the miR-30 family promotes invasion and migration.^[27]^

While there are many miRNAs that promote resistance in PDAC, there are also many other miRNAs that enhance PDAC chemosensitivity. Typically, expression of these miRNA is reduced in PDAC and restoration of their expression helps to combat chemoresistance. miR-34 is a well-studied tumor suppressor miRNA that is regulated by p53. In PDAC loss of miR-34 is associated with enrichment of stem like cells, while restoration of miR-34 inhibits growth and enhances sensitivity to gemcitabine.^[28,29]^ Similar to miR-34, miR-205 has also been shown to inhibit PDAC stem cells and promote sensitivity to gemcitabine.^[30]^ Various studies have also shown that the miR-200 as well as the let-7 family are reduced in gemcitabine resistant cells and expression of these miRNAs promote chemosensitivity.^[31]^ Several groups have also demonstrated the importance of miR-506 in combating chemoresistance in PDAC. Through the regulation of several different targets, miR-506 promotes apoptosis, induces cell cycle arrest and enhances chemosensitivity.^[32,33]^ miR-15a is another miRNA with important roles in PDAC resistance. miR-15a suppresses the growth of chemoresistant PDAC cells and inhibits EMT by targeting WNT3A, FGF7 as well as BMI-1.^[34,35]^ These are just some examples of the important functions of miRNAs in PDAC resistance (Table 1). Clearly depending on the miRNA, it may either help to promote or combat resistance. Either by inhibiting onco-miRs or reintroducing tumor suppressor miRNAs, we can leverage these functions for therapeutic effects.

## Therapeutic potential of miRNAs in PDAC

There are several strategies to develop miRNA-based therapeutics in PDAC. One is to suppress onco-miRNAs via antisense or antagomir-based approaches, the other is to restore and replace tumor suppressor miRNAs.^[36]^ Because miRNA can impact of multiple targets and pathways, miRNA-based therapy will have a potential to overcome the complex resistance mechanism in cancer.^[37]^ However, with regard to the therapeutic development, there are several major hurdles (e.g., delivery and poor pharmacokinetics) that needs to be overcome.^[38]^ A number of strategies have been developed to overcome such hurdles. Various nano-vehicles and nanoparticles have been developed and tested.^[39–41]^ However, many of these delivery vehicles cause host toxicity. Various improvements have also been developed to enhance stability of anti-miR by modification of the backbone with 2′-*O*-methyl group, phosphothioate, or with the locked nucleic acid.^[42]^

**Figure 1.**
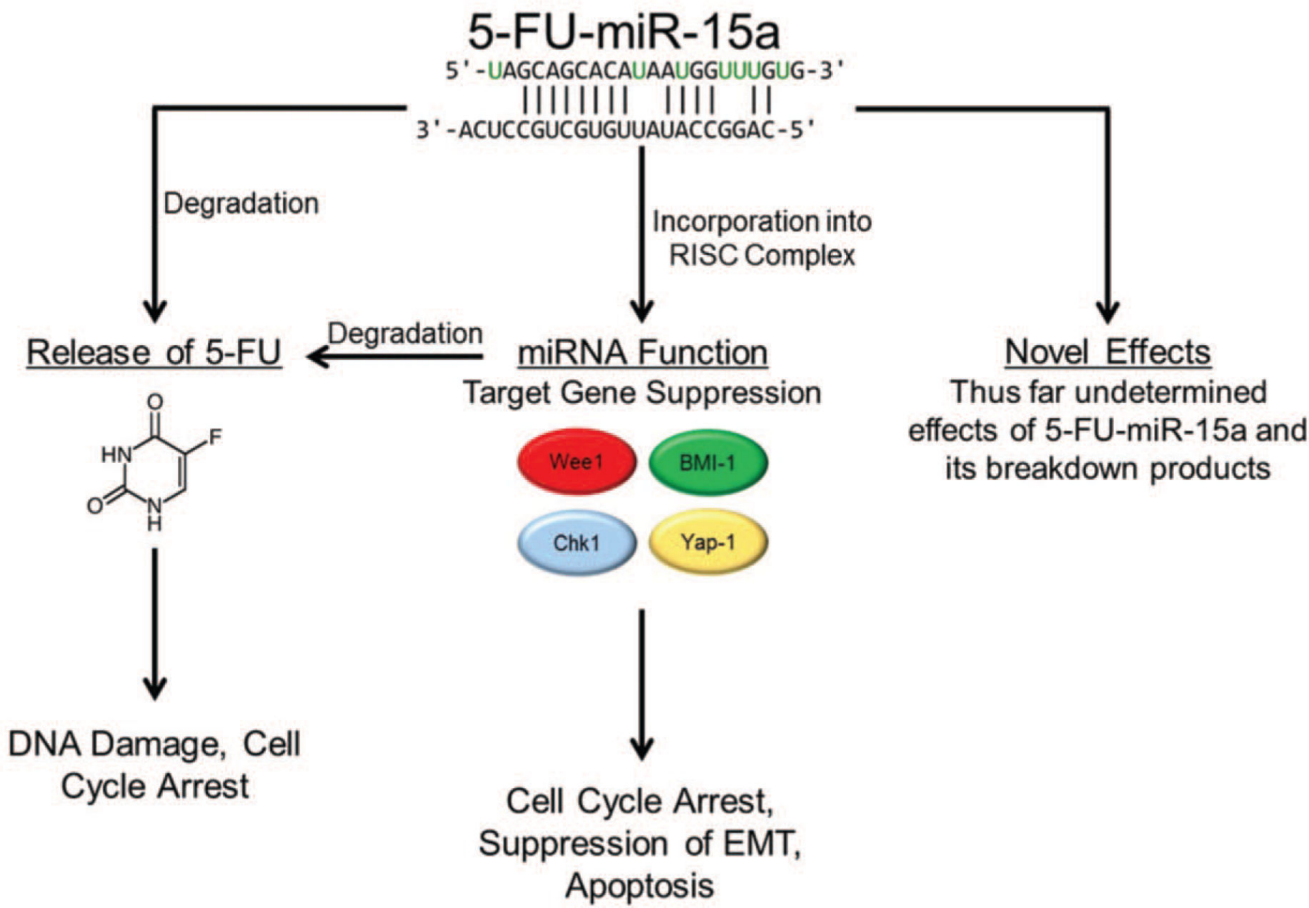
5-FU-miR-15a combined the tumor suppressive effects of miR-15a with those of 5-FU. 5-FU-miR-15a is incorporated into the RISC complex resulting in suppression of miR-15a’s target genes, Wee1, Chk1, BMI-1, and Yap-1. Suppression of these targets induces cell cycle arrest, apoptosis, and suppresses EMT. When the 5-FU-mIR-15a breaks down 5-FU is released which will lead to DNA damage and cell death. In addition the modification of the miRNA may have some novel effects resulting from the unique modification as well as its breakdown products. Chk1 = checkpoint kinase 1, EMT = epithelial-to-mesenchymal transition, 5-FU = 5-fluorouracil, miRNA = microRNA, RISC = RNA-induced silencing complex.

We have recently developed a novel miRNA replacement strategy by integrating the therapeutic power of chemotherapeutic agents such as 5-FU with tumor suppressor miRNAs in PDAC. It is not just a simple combination of 5-FU with tumor suppressor miR, as 5-FU alone can still have major side effects in patients, but rather, we incorporate 5-FU into tumor suppressor miR by replacing Uracil (U) with 5-FU in the guide strand. We initially developed this approach using miR-129 and miR-15a in colon cancer.^[43,44]^ By incorporating 5-FU into miR-129 and miR-15a, we created a potent new miRNA-based drug with the power of tumor suppressive function of miRNA together with 5-FU (Fig. 1). We demonstrated these modified miNRA have some unique features such as enhanced stability and potency, with no observed host toxicity. 5-FU-modified miRNA can also be delivered to tumor cells vehicle free. This is a major advancement that may help to overcome the bottleneck of delivery that hampers nucleic acid-based therapeutics.

Our recent studies demonstrated miR-15a functions as a tumor suppressor in PDAC *in vitro* by inhibiting cell proliferation and impacts cell cycle control.^[45]^ We have demonstrated that 5-FU-modified miR-15a is a potent inhibitor for PDAC both in vitro and in vivo and improves survival, either alone or in combination with gemcitabine. The effects of miR-15a are mediated through the regulation of several important target genes (*Wee1*, checkpoint kinase 1 [*Chk1*], *BMI-1*, and *Yap-1*). All of these targets are elevated in PDAC and many are good target candidates for therapeutic development in PDAC. B lymphoma Mo-MLV insertion region 1 is an oncogene associated with poor prognosis.^[46,47]^ Yes-associated protein 1 is crucial in promoting pancreatic tumorigenesis as well as invasion, migration, and chemosensitivity.^[48–50]^ Wee1 and Chk1 are 2 key G2/M checkpoint regulators, which can affect Cdc2 activity.^[51,52]^ These targets have been recognized as candidates for therapeutic development by the pharmaceutical industry.^[51,53,54]^ The regulation of all of these important targets combined with the effectiveness of the 5-FU incorporation makes 5-FU-modified miR-15a an intriguing candidate for PDAC therapy.

In addition to reintroducing tumor suppressor miRNAs, suppression of onco-miRNAs also has potential. By inhibiting these miRNAs expression of their target genes can be restored which may have therapeutic effects. A recent study has demonstrated the potential of anti-miR-21. Delivery of antimiR-21 was shown to be effective in patient derived PDAC organoids and patient derived xenograft models using tandem peptide pTP-iRGD.^[55]^

## Conclusion

There are many mechanism that contribute to both the intrinsic and acquired resistance seen in PDAC. The adaptive nature of these cells makes overcoming resistance a moving target. This is highlighted by the fact that efforts focusing on single protein coding targets have yielded little progress on PDAC drug development in the past decades. In the face of this challenge, miRNA therapeutics may be excellent therapeutic candidates. Their ability to quickly modulate the expression of multiple targets gives them the unique ability to regulate several pathways at once. This should make it more difficult for cells to adapt and overcome miRNA therapy. As a result, a number of miRNAs have shown great potential as novel therapeutics in PDAC. With advancement in miRNA research and therapeutic development, we expect that tumor suppressive miRNAs, including 5-FUmodified miRNAs will allow us to better manage clinical treatment and enhance survival and minimize toxicity for PDAC patients.

## Figures and Tables

**Table 1:** miRNAs with roles in chemoresistance.

miRNAs that promote chemoresistance		miRNAs that promote chemosensitivity	
miRNA	Target gene	miRNA	Target gene
miR-21^[20,21]^	PTEN,^[20]^ RECK^[20]^	miR-34^[26,27]^	Notch,^[27]^ BCL2^[27]^
miR-320a^[22]^	PDCD4^[22]^	miR-205^[28]^	ZEB1^[28]^
miR-221–3p^[23]^	RB1^[23]^	miR-200^[29]^	ZEB1^[29]^
miR-106a^[24]^	TP53INP1^[24]^	miR-506^[30,31]^	SPHK1,^[30]^ PIM3^[31]^
miR-155^[25]^	TP53INP1^[25]^	miR-15a^[32,33,44]^	WNT3A,^[32]^ FGF7,^[32]^ BMI-1,^[33,44]^ Wee1,^[44]^ Chk1,^[44]^ Yap-1^[44]^

Chk1 = checkpoint kinase 1, miRNA = microRNA.
